# Clinical governance and research ethics as barriers to UK low-risk population-based health research?

**DOI:** 10.1186/1471-2458-8-396

**Published:** 2008-11-28

**Authors:** Edwin R van Teijlingen, Flora Douglas, Nicola Torrance

**Affiliations:** 1 Section of Population Health, School of Medicine & Dentistry, University of Aberdeen, Aberdeen, UK; 2 School of Health & Social Care, Bournemouth University, Bournemouth, UK; 3 Centre for Academic Primary Care, School of Medicine & Dentistry, University of Aberdeen, Aberdeen, UK

## Abstract

**Background:**

Since the Helsinki Declaration was introduced in 1964 as a code of practice for clinical research, it has generally been agreed that research governance is also needed in the field of public health and health promotion research. Recently, a range of factors led to the development of more stringent bureaucratic procedures, governing the conduct of low-risk population-based health research in the United Kingdom.

**Methods:**

Our paper highlights a case study of the application process to medical research ethics committees in the United Kingdom for a study of the promotion of physical activity by health care providers. The case study presented here is an illustration of the challenges in conducting low-risk population-based health research.

**Results:**

Our mixed-methods approach involved a questionnaire survey of and semi-structured interviews with health professionals (who were all healthy volunteers). Since our study does not involve the participation of either patients or the general population, one would expect the application to the relevant research ethics committees to be a formality. This proved not to be the case!

**Conclusion:**

Research ethics committees could be counter-productive, rather than protecting the vulnerable in the research process, they can stifle low-risk population-based health research. Research ethics in health services research is first and foremost the responsibility of the researcher(s), and we need to learn to trust health service researchers again. The burden of current research governance regulation to address the perceived ethical problems is neither appropriate nor adequate. Senior researchers/academics need to educate and train students and junior researchers in the area of research ethics, whilst at the same time reducing pressures on them that lead to unethical research, such as commercial funding, inappropriate government interference and the pressure to publish.

We propose that non-invasive low-risk population-based health studies such as face-to-face interviews with health and social care professionals or postal questionnaire studies with patients on non-sensitive topics are given a waiver or a light touch review. We suggest that this can be achieved through a two-staged ethics application process. The first stage starts with a one or two-page outline application which ethics committees can use as the basis to grant a waiver or request a full application.

## Background

There is a general agreement that more and better quality research is needed to develop evidence in the field of public health and health promotion [1]. However, a range of factors have led to the development of more stringent bureaucratic procedures, governing the conduct of health services and health promotion research in the UK [2,3]. These procedures are partly as result of EU (European Union) legislation for clinical research, such as the Data Protection Act and Human Rights legislation, and have resulted in (a) standardization of procedures [4]; and (b) additional challenges in conducting low-risk population-based health research [5]. At a more abstract level some have argued that the increased 'control' of health research is part of a trend in society, which as a whole is becoming more risk averse [6,7].

Our paper highlights a case study of the application process to medical research ethics committees in the UK for a study of the promotion of physical activity by health care providers [8]. Our case study in public health presented here is an illustration of the challenges in conducting public low-risk population-based health research in the UK. Our mixed-methods study consisted of a questionnaire survey of and semi-structured interviews with primary care professionals. All professional were volunteers and as we did not conduct research with the general population, one would expect the application to the research ethics committees to be a formality. Before we introduce our case study we will consider the origins of medical and health research ethics committees.

### Origins of research ethics committees

In 1964 the World Medical Association drew up the first Declaration of Helsinki (see most up-to-date version at ), the most widely accepted guidance on biomedical research on humans; its key principles are listed in Table 1. This Declaration was a response to medical experiments conducted on prisoners in Nazi concentration camps across Europe during the Holocaust [9,10], as came to light during the Nuremburg trials. It is now generally accepted that research participants have a right to expect that the research is conducted to appropriate standards, and adds something to the body of knowledge as highlighted in the Nuremburg Code of 1947. Although serious research misconduct is relatively rare, the exceptions remind us that vigilance is always required, especially because misconduct can be hard to prove [11]. One of the textbook examples of serious health services research misconduct is the Tuskegee Syphilis Study [10]. Between 1932 and 1972 researchers in a long-term study regularly examined a large number of poor African Americans in Alabama (USA), suffering from syphilis, to study the natural course of the disease [12]. These men were made to believe that they were receiving proper medical treatment when, in fact, either no treatment or inadequate treatment was given.

**Table 1:** The four key ethical principles

Do no harm/non-maleficence
Do good/beneficence

Justice

Respect for autonomy

These extreme cases highlight why we need a public body to protect the general population from unethical research. In the Declaration of Helsinki, the World Medical Association [13] outlined that a research protocol "should be submitted for consideration, comment, guidance, and where appropriate, approval to a specially appointed ethical review committee, which must be independent of the investigator, the sponsor or any other kind of undue influence." The reply in the USA has been the establishment of IRBs (Institutional Review Boards) at universities and research institutes, whilst the UK originally went down the path of local NHS (National Health Service) research ethics committees (REC), although many British universities have began to establish their own RECs for health research conducted outwith the NHS. For example, the one for our own university was established in 2008.

There have been considerable changes in the past decade in the rules and regulations governing applications for ethical approval to conduct low-risk population-based health research in the UK [14]. New national research governance frameworks are intended to improve the research process. These changes are supposed to make applying for research ethics permission more transparent, with a greater emphasis on ethical considerations for researchers. In practice, the changes introduced in the past few years have made the process of applying for ethical approval more time-consuming, complex, and bureaucratic [15-17]. The standard application form for MRECs (Multicentre Research Ethics Committees) and/or local RECs is long and fairly complicated. For example, the NHS REC application form in use in the summer of 2007 was 80-odd pages long, and some point in 2005 it was as long as 96 pages.

This paper reports on the process of applying for research ethics approval for a relatively straightforward, unobtrusive, non-interventionist health promotion study. It highlights some of the oddities of the current UK system, and the potential impact on the ability to conduct good quality health promotion and health education research in the future. We suggest some improvements to the current system of gaining research ethical approval for low-risk health and health promotion research, which are based on principles of pragmatism, consistency and efficiency, as well as the four key principles listed in Table 1

#### Ethics committees in the UK

In the UK health service, clinical, health care, public health, nursing, epidemiological, and health promotion research must be considered under general biomedical research ruling [18]. Each region has one or more separate research ethics committees which operate independently from the local universities and health-care providers. A national body called COREC (Central Office for Research Ethics Committees), was established by the UK Government to oversee all the area-based research ethics committees. This was replaced in 2008 by the National Research Ethics Service (NRES) run by the NHS National Patient Safety Agency . Therefore, anybody conducting research in the UK on NHS premises, with NHS patients and/or NHS staff has to apply to such a committee. In theory then all applications nationwide are assessed by the same criteria by a similar committee. It is universally accepted that a team of doctors wishing to conduct a clinical trial of a pharmaceutical drug with potentially dangerous side effects must apply for appropriate ethical approval. However, it is also the case that social or health researchers planning a study, of for example, NHS hospital porters' attitudes towards cycling to work must apply to the same research ethics committee.

There are around 200 local RECs in the UK, each with a mixture of between 10 to 20 (lay and professional) members [19]. The remit of a research ethics committee is "to protect the rights, safety, dignity and well being of all actual or potential participants" [20]. The role of each committee is to assess the potential ethical implications of the proposed research as outlined on the application form, the study protocol. In addition the study materials are considered, for example, the participants' information sheets, letters inviting people to participate in a study, posters to recruit interviewees in a clinical setting and any other items submitted. The process of applying for ethical approval is widely considered to be a lengthy, bureaucratic one [21]. If a research project fails to receive ethical approval the proposed research should not go ahead until a revised proposal which addresses the concerns of the REC is accepted.

Problems have arisen in this system when researchers have looked to conduct a nationwide study or a study involving participants from more than one region. Hence Multi-centre Research Ethics Committees (MRECs) were introduced to avoid researchers having to apply to several local RECs with the same application [18,22]. At the time of our study a two tier-system existed, whereby MRECs, as overarching ethics committees, considered the key ethical issues of the proposed research, whilst the second tier, the RECs, considered specific local issues, such as, for example, the number of studies already running locally in the same patient population in order to avoid overburdening these participants.

Over the past two decades there has been much criticism from the research community (and not just in Public Health) on the way RECs operate in the UK [4,15-17,23-30]. Barker, for example, describes an epidemiological study of a pre-NHS archive of health visitors' records babies living in Hertfordshire (UK) [31]. Based on these records Barker and his team conducted a long-term epidemiological follow study of 15,000 people some 50 years later. His final comment is highly relevant here:

At the time we had no difficulty in getting permission to trace, interview, and examine large numbers of people. It is unlikely that such permission would be so readily obtained today. Had current data protection laws been in force 15 years ago, they might have prevented thousands of willing Hertfordshire people from taking part in medical research-and the fetal origins hypothesis would not exist [32].

Of course, the RECs system is not fail-safe; even with active ethics committees there is still going to be unethical research. For example, having undergone an appropriate ethical review did not prevent the UK scandal of body parts of children being kept for research without parents being informed or asked for permission at Alder Hey Hospital in Liverpool [33]. Whilst the 2006 drug trial at Northwick Park Hospital (London), that caused serious side effects in six healthy male volunteers in a private facility [34], had been reviewed and approved by the appropriate research ethics committee. As Dingwall reminded us, the relatively regulatory environment in Germany of the 1930s did not prevent the medical experimentations of the Nazi in the 1940s [34]. So although we are convinced of the importance of having such ethics committees, it is clear that they can not (and will not) police all on-going research to help maintain ethical standards. Therefore, it is ultimately a key role for researchers to consider the ethical elements of their research (and that of their students), and to ensure that research is being conducted ethically.

### Case study

Our case story highlights an ethics application process which took place recently (2004) and raises a number of ethical and practical public health research issues for low-risk population-based research. We were successful in winning a research contract (after a competitive tendering process) to conduct a national study to investigate knowledge, attitudes and current practice amongst primary care staff associated with promoting physical activity as an aspect of routine patient consultation. Primary care staff in this study refers to family doctors/general practitioners (GPs), health visitors, and practice nurses, and our study did not involve vulnerable groups, NHS patients, or the general public [8]. It was solely directed at health care professionals.

We planned to use a mixed methods approach, including a postal questionnaire in four health regions, and interviews with a sub-sample of GPs, practice nurses and health visitors. We aimed to conduct the majority of the staff interviews by telephone in order to minimise the disruption to their work time. All interviewees were volunteers. The kind of questions asked were not personal in terms of addressing respondents' life or lifestyle, but related, for example, to the advice given on physical activity to their patients/clients. Additional file 1 lists examples of the kind of questions we addressed to convince the reader that the questions in questionnaire did not raise any obvious ethical concerns, neither was there any probing into health professionals' personal life.

### MREC application

The initial ethics application was made to MREC using the 68-page form in use at the time, and endorsement was granted within six weeks. Although the MREC did not ask us to change any aspect of our study, it informed us that we should not start the any part of it until we had LREC endorsement from the four regional health board areas concerned.

Having sent the required number copies of the application with similar number of copies of supporting documentation to the MREC, their response letter to us stated that each local REC should receive a copy of the MREC letter and response form and Part C of the COREC form plus similar number copies of questionnaire.

The following sections outline the quite different responses from the four local RECs. We have labeled them A-D to avoid identification. REC A replied quite quickly (within three weeks) to inform us that we could proceed in their area as soon as we wanted. So far, so good.

The second REC took longer to reply (5 weeks) and gave approval on the basis that they considered there were no locality issues relating to the application. However, REC B did draw our attention to minor changes in the study materials (which the MREC had not commented on); required us to seek local NHS management approval and give guarantees on medical indemnity. REC C was experiencing an organisational restructuring at the time and after some negotiating, the acting Director of Public Health granted management approval for the study to go ahead on the basis that MREC approval had already been granted.

We had to follow-up REC D as we had received no communication after two months. Our prompting resulted in a letter explaining that one of the committee members had concerns about confidentiality and anonymity issues and storage of data – an issue which had not been raised by MREC, whose primary role (distinct from the LREC) is to assess all ethical issues in our multicentre study. In addition, we were required to gain the endorsement of the local Primary Health Care Trusts Research & Development committee. Such committee manages all research with implications for the NHS. REC D also insisted on receiving a copy of our final report. In addition to sending the documentation as advised by MREC (see above), we were also required to submit copies of the CVs of all researchers, a copy of the study protocol, the REC letter and our response regarding their concerns about confidentiality. Additionally we were required to complete and sign a Data Protection Checklist.

In the end we gained all the necessary approval from all four RECs five months after lodging the original application with MREC. Unnecessary delays 'caused' by the length of time taken by research ethics committees to come to a decision can, of course, add to the cost of a research project.

## Discussion

We start the debate with issues arising from our case study. This is followed by a wider discussion of the ethical approval and ethics committees and the implications for Public Health research in general. It is clear that the four RECs dealt differently with our application. We had expected minor variation, based on local circumstances, but not major procedural differences. RECs A and C did not appear to re-review the research application in line with their remit. REC B came back to the research team with minor observations, which suggests that it had made ethical considerations where it should only have considered local circumstances. Its request for guarantees on medical indemnity is standard practice in clinical research, but perhaps surprising (unthinking) as our study did not involve patients at all. Getting ethical approval from REC D was most cumbersome as it raised issues apparently outwith their remit.

For a study which did not get close to patients, the process of applying for research ethics permission took five months and a lot of work! This extra administrative burden was both time-consuming and frustrating to the researchers (and the funders).

### Practical considerations

Our experience highlights several problems inherent in the current system of area based RECs. First, we found that local ethics committees operate in very different ways. Several critiques in the UK have reported a lack of standardization between research ethics committees in the ways they process applications [27,35,30]. Many more have criticized the inconsistency in outcome to the same application between different RECs [25,26,36]. Unfortunately, we have to agree with Hannigan and Allen [37] that it is "not clear how far the new UK research governance framework will help to reduce the unpredictability of REC decision-making." Moreover, we felt (as a research team with a small budget, and a strict time scale to pursue) powerless to do anything about how we were being dealt with, in case it subsequently jeopardised our ability to conduct the project in the time we had available. We are aware that inconsistencies in ethical considerations are not just a UK problem, but also a cross-national one. For example, Glasziou and Chalmers reported that piloting of a leaflet aimed at older people to improve GP consultations needed ethical review, in Belgium, Slovenia and the UK, but not in Austria, France Germany and Switzerland [22], whilst the UK has one of the most complicated and arduous processes [38].

The second consideration concerns resources, especially staff time needed to complete the application process. We had believed that taking the time initially to seek MREC approval would help to speed up the process – but this did not appear to be the case. Furthermore, there are opportunity costs related to the staff time and salaries required to consider and respond to applications. A burden borne not only by the research community, but also by the 'cash-strapped' health service. Interestingly, only a small proportion of researchers in the study by Richardson and McMullan [39] commented "that research opportunities were being lost through the time taken to obtain ethical approval". Moreover, no one seems to mention the time of the endless number of volunteers across the UK on MREC, LRECs, IRBs and NHS Research & Development committees. RECs can add to the workload of researchers, and hence the cost of research. One research group calculated that their ethics application took two weeks to complete and required 44.5 hours of activity at an estimated cost of £850 [2]. In another example, in one UK clinical trial, researchers had to submit between one and 21 copies to each of 125 ethics committees [40].

We also found that the division of labour and jurisdiction between the MREC and the LRECs was not a distinct as the regulations suggested. We found that in reply to the same proposal several LRECs requested that changes were made on issues which were the jurisdiction of the MREC, not of the individual LREC.

### Academic research considerations

At a wider societal level there are concerns that ethical governance stifles certain types of low-risk population-based, epidemiological, health and social service and public health research. A classic example case was made by Sir Richard Doll [41], who argued that the case-control study in the late 1990s on the link between induced or spontaneous abortions and breast cancer [42] would no longer be possible in the new Millennium. His reason for saying this relates to the limitations introduced by the 1999 Data Protection Act which requires that researchers ask patients for permission to use data collected for a different purpose. Sin reported on a social science study of older people from different ethnic minority groups in Britain. This study was also caught by the introduction of the Data Protection Act when the relevant UK government body was no longer permitted to provide a sample [43]. Ethics committees are directed by the Data Protection Act and similar legislation for guidance on ethics applications in the clinical research field, which, partly due to standardization, also cover all other health research.

Professional and academic researchers are currently feeling challenged and beleaguered in their endeavors to secure research funding and ethical approval [3,4,34,41]. Although there has long been a demand by UK researchers for improvements in the way RECs operate [26,27,44], recent changes in the process have not been seen as an improvement. We would like to highlight recent changes made by the National Health and Medical Research Council in Australia in their *National Statement on Ethical Conduct in Human Research 2007 *which offers different guidance for "very different types of research" [45]. This Australian national statement suggests that ethics committees may establish procedures for expedited review of research involving minimal risks to participants. It is unclear how this is going to work in practice, but it incorporates some of the worries we have highlighted in this debate paper.

### The researcher is central!

Although research ethics committees can reduce the risk of unethical research being conducted, they can not prevent it as the various ethical misconducts mentioned above indicate. We argue that the researcher is central to the ethical research process. Thus we fully agree with Hannigan and Allen [27] that ultimately the responsibility to ensure that studies are ethically sound lies, where it has always been, namely with the researchers. Or as Holmes put it: 'We must trust researchers- as we do physicians and surgeons 'to do the right thing' [46]. Hence researchers need to be competent in at least two respects: (1) they must have research skills; and (2) must be sufficiently competent to care for the subject [10]. Therefore, we must educate our students and junior researchers in research ethics so they too can be trusted to do the right thing.

### Bureaucratisation of the research process

Ethics committees seem to be expanding their scope and remit to cover more and more previously uncovered ground [34]. Making research ethics a bureaucratic tick-box process can be counterproductive [43,47]. As Barbour highlighted in her argument against the use of checklists in qualitative research as we may find we are ending up with the tail wagging the dog [48]. Or as Richardson and McMullan noted, it would appear that the UK system of research ethics governance is in danger of throwing the baby out with the bath water [4], whilst Dingwall put it even stronger by suggesting that ethical regulation of low-risk social research exceeds the harm that the actual research could do to its participants [49].

One has to ask the question, 'What is the wider societal problem?' Is there societal demand for increased "safe guards" to protect against potentially harmful/inappropriate research studies and/or researchers? Or is this a reflection of inappropriate arduous legislation translation of the Declaration of Helsinki. Or is it part of a general trend towards increasing societal control in the UK in general, including research, e.g. anti-terrorism legislation re. the call to make everyone carry a national identity cards, or police checks for every parent volunteer at their child's school football tournament?

We propose that non-invasive observational studies, i.e. low-risk population-based health research, including face-to-face interviews with health and social care professionals or postal questionnaire studies with patients on non-sensitive topics are given a waiver or a light touch review. Perhaps this can be achieved by the introduction of a two-staged ethics application process. First, a preliminary stage with a short one or two-page outline application which LRECs can use as the basis to grant a waiver, and secondly a full application for those projects not deemed to be at low-risk. Drug companies and researchers planning to conduct invasive interventions may skip the preliminary application and proceed directly to the full-application stage.

Ethical regulation has become cumbersome and time consuming [21], and starts to act as a barrier to low-risk population-based health research and the progress of expanding our knowledge in this field. Knowledge, which as Cochrane reviews (see: ) often tells us, is often lacking for community-based health promotion and public health interventions. We would like to share our experiences with readers of this journal to stimulate a debate, with the aim of drawing attention to the plight of those engaged in low-risk public health research and evaluation – which must be conducted in line with research governance guidelines – but at the same time, is often done so in the context of scant or no resources.

## Summary

1. Having one general research ethics application form originally designed for clinical and pharmacological research for all types of low-risk research, leads to over-regulation and the inappropriate application of a biomedical framework.

2. Balance the needs of researchers in their quest to conduct high quality research and the protection of individual study participants, as applying rigid approaches to research ethics guidelines could severely restrict progress in vital population-based studies.

3. Ethics committees should appraise the risk from research projects aimed designed for (a) individual patient-based pharmaceutical and surgical interventions; (b) population-based, anonymous health and social services and health promotion research; and (c) studies of ordinary people, (i.e. non-patients) and staff.

4. Non-invasive low-risk studies such as face-to-face interviews with health and social care professionals or postal questionnaire studies with patients on non-sensitive topics are given a waiver or a light touch review. This can be achieved by a two-staged ethics application process, a preliminary stage with a short outline application on the basis of which LRECs can grant a waiver or request a full application.

5. It is important to train junior researchers and students about their role in setting and maintaining ethical standards in research.

## Competing interests

The authors declare that they have no competing interests.

## Authors' contributions

All three authors were actively involved in the primary study that gave rise to the ethical issues raised in this debate paper. EvT conceived the paper. All authors discussed the ethical issues and implications. EvT and FD designed the structure of this paper. All authors read and commented on various drafts and all approved the final manuscript.

## Pre-publication history

The pre-publication history for this paper can be accessed here:



## Supplementary Material

Additional file 1 **Appendix.** Two sample study questions. Click here for file
