# Supplementary material for: Clinical governance and research ethics as barriers to UK low-risk population-based health research?
Source: BMC Public Health. 2008 Nov 28;8:396. doi: 10.1186/1471-2458-8-396 (PMC2612000; doi:10.1186/1471-2458-8-396)
Supplement: Additional file 1 — Appendix. Two sample study questions. [file 1471-2458-8-396-S1.doc]

Appendix

Two sample questions from our study

1. What are the current physical activity recommendations for inactive/not regularly active, apparently healthy adults?

1. During consultations with adult patients who are apparently healthy, which of the following activities are you likely to perform in relation to physical activity?

Please place a tick () after every statement.

|  | *Very likely* | Likely | *Unlikely* | Very Unlikely |
| --- | --- | --- | --- | --- |
| 1. Discuss health benefits of physical activity | 🞏 | 🞏 | 🞏 | 🞏 |
| 1. Discuss psychological benefits of physical activity | 🞏 | 🞏 | 🞏 | 🞏 |
| 1. Advise patients to be more physically active by doing housework, heavy gardening etc. | 🞏 | 🞏 | 🞏 | 🞏 |
| 1. Advise patients to walk more as part of daily activities | 🞏 | 🞏 | 🞏 | 🞏 |
| 1. Advise patients to participate in moderate exercise (exercise that causes you to be warm, slightly out of breath and makes you heart beat faster than normal). | 🞏 | 🞏 | 🞏 | 🞏 |
| 1. Advise patients to participate in vigorous exercise (exercise that causes you to sweat, breathe harder and makes your heart beat fast). | 🞏 | 🞏 | 🞏 | 🞏 |
